# Prevalence and antimicrobial susceptibility of gram-negative bacteria in the urine of females in their reproductive ages in the Tamale Teaching Hospital

**DOI:** 10.1186/s41043-025-00853-y

**Published:** 2025-08-04

**Authors:** Rashida Ibrahim, Abudu Ballu Duwiejuah, Kennedy Mensah Osei

**Affiliations:** 1 Department of Biotechnology and Molecular Biology, Faculty of Biosciences, University for Development Studies, Tamale, Ghana; 2 Laboratory Department, Tamale Teaching Hospital, Tamale, Ghana; 3 College of Medicine, Yonsei University, Seoul, South Korea

**Keywords:** Bacterial antimicrobial susceptibility, Gram-negative bacteria, *Klebsiella* spp, Urinary tract infection, Ghana

## Abstract

Regardless of where the illness occurs in the urinary tract, a urinary tract infection (UTI) is defined as the presence of substantial bacteria in urine. The objective was to determine the prevalence of UTIs among females in their reproductive age group (15–45 years) in Tamale Teaching Hospital. A total of 132 samples were collected for urine culture and sensitivity from January to April 2022. The urine samples were analysed using biochemical methods. The isolated bacterial pathogens were identified using standard bacteriological methods. In the 132 urine samples analysed, a higher frequency of bacteria was observed in the age group 21–25 years, followed by the age group 26–30 years, and then 15–20 years. *Klebsiella* spp had a prevalence rate of 30.95% followed by *E. coli* (23.81%), *Klebsiella oxytoca* (14.29%), *Acinetobacter baumannii* (7.14%), *Citrobacter freudii* (2.38%), *Pseudomonas aeruginosa* (2.38%) and the other gram-positive organisms put together had 19.05%. *Klebsiella* spp was the most dominant bacteria pathogen causing UTIs among females. The isolated bacterial pathogens were most sensitive to Meropenem (MRP) and Levofloxacin (LVX). *Acinetobacter baumannii* was the only isolated bacteria pathogen resistant to MRP. Based on the study’s outcomes, LVX and MRP are suggested as first-line treatments for UTIs whilst doctors wait for culture and sensitivity test results from the lab.

**Supplementary Information:**

The online version contains supplementary material available at 10.1186/s41043-025-00853-y.

## Introduction

Urinary Tract Infection (UTI) is the presence of significant bacteria in urine irrespective of the site of infection in the urinary tract (Osungunna and Adenike 2017). Whilst kidney and ureter infections are a sign of an upper urinary tract infection, bladder and urethral infections are referred to as lower urinary tract infections. Urinary tract infection affects people of both sexes and all age groups [16]. 50% of women experience at least one episode of UTI throughout their lifetime due to anatomical posture, physiological changes, vaginal activity, the use of contraceptives like spermicide and the diaphragm, and a lack of prostatic fluid, which functions as an antibacterial agent [42]. Even though men get UTI episodes less frequently than women do, they are nevertheless more dangerous when they do [29]. Despite these higher UTI risks, doctors lack reliable scientific tools for detecting and eventually treating UTI complaints in patients. As a result, UTIs can result in major side effects such as repeated recurrences, bacteremia, renal failure, and premature birth [15].

Antibacterial medications are useful for treating bacterial infections. Although some bacteria are naturally resistant to even recently developed anti-bacterial drugs, the majority of pathogens have been found to develop acquired antimicrobial resistance [44]. An antimicrobial is a substance that either eliminates or inhibits the growth of bacteria. Antimicrobials have been used to protect the human population from the threat of infectious diseases and are one of the most effective types of chemotherapy (Das and Patra 2017). Gram-negative bacteria are those that lose their crystal violet colour when subjected to the Gram-staining procedure. Gram-negative bacteria include, among others, *Escherichia coli*, *Klebsiella* species, *Pseudomonas* species and *Proteus* species. Most gram-negative bacteria are non-spore-producing bacilli that proliferate quickly in both aerobic and anaerobic environments on common laboratory media [39].

Antibiotic-resistant bacteria are a global public health concern that can result in treatment failure, high treatment costs, and increased morbidity and mortality. However, indiscriminate use and uncontrolled access are causing microorganisms to become resistant to treatment [47]. Most patients with suspected UTIs visit neighbourhood drug stores managed by unskilled staff to buy medications out of frustration, which promotes antibiotic resistance and unsuccessful treatment [47]. Due to inadequate healthcare delivery infrastructure and a lack of training in the correct management of infectious diseases, Ghana has a very high prevalence of bacterial infections, and the treatment of these infections is typically quite poor. The majority of uncomplicated UTI infections can be mild and short-lived; nevertheless, if left untreated, UTIs can cause renal scarring, hypertension, and eventually end-stage renal disease. It is crucial to diagnose UTIs correctly and promptly. Since it is spreading from one region to another, antibiotic resistance is a problem that affects the entire world. Therefore, for effective treatment, professionals require some background knowledge, such as the most common bacteria that cause UTIs and their susceptibility to antibiotics. The study was to determine the prevalence of UTIs among females in their reproductive age group at the Tamale Teaching Hospital.

## Materials and methods

### Study area

The study was conducted at Tamale Teaching Hospital. Tamale Teaching Hospital is located on Salaga/Yendi Road. After Korle Bu Teaching Hospital and Akomfo Anokye Teaching Hospital, it is the third teaching hospital in Ghana.

### Study population

The study population consisted of females of child-bearing potential whose age ranges from 15 to 45 years old. They were referred for urine culture at Tamale Teaching Hospital during the study period and who did not initiate antibiotics therapy during the last two weeks.

### Sampling and data collection

The study used the purposive sampling technique to select females of their reproductive ages referred for urine culture and antibiotic susceptibility tests at Tamale Teaching Hospital. The researchers met with the lab's clinicians and nurses before the study began and went through the details of the protocol. The nurses selected females in their reproductive age (15–45 years) who were referred for urine culture and antibiotic susceptibility tests and also who met the inclusion criteria, and demonstrated to them how to collect the urine sample. The study’s participants were chosen until the target sample size of 132 was reached. A consent form was administered.

A cross-sectional study was conducted from the beginning of January to April 2022 on the prevalence and antimicrobial susceptibility of gram-negative bacteria in the urine of females in their reproductive ages in the Tamale Teaching Hospital. A total of 132 samples were collected for urine culture and sensitivity. To access the prevalence and sensitivity pattern of the urinary pathogens, midstream urine samples obtained in January were investigated using cultural methods. This whole process was carried out in the Bacteriological laboratory at Tamale Teaching Hospital. Urine microscopy, culture, and sensitivity were requested to detect or indicate a urinary tract infection linked to gram-negative organisms.

### Inclusion and exclusion criteria

The inclusion criteria for the study were females of the reproductive age group (15–45 years), who were healthy and willing to participate in the study.

Females receiving any sort of antibiotic therapy and females whose families refused to sign consent forms were both excluded from the study.

### Informed consent form

This study was carried out after obtaining approval from the University for Development Studies Institutional Review Board (UDSIRB) [UDS/RB/032/21]. The study was performed by the ethical standards laid by the University for Development Studies. The consents of the patients or hospital attendants were verbally sought which consented and confidentiality and anonymity were assured before sample collection. The purpose of the study was explained to females and their right to withdraw from the study at any point in time. Females were given 10 mL of early morning urine specimen for analysis.

### Urine culture

The media of choice for urine culture is Cysteine Lactose Electrolyte Agar (CLED). Urine samples were thoroughly mixed and the container opened. A sterile 10 µl bacteriological loop was inserted in each urine sample vertically and allowed urine to adhere to each loop. A loopful of urine was spread over a CLED agar plate using the standard method. Plates were incubated aerobically at 35–37 °C for 18–24 h. On the next day, the bacterial growth on the respective media was looked at, and total colonies were calculated by multiplying by 100 (since a 0.01 mL loop was used). Antibiotic susceptibility testing was then carried out.

### Gram staining

A colony was selected from a plate using a flamed wire loop, and a thin film smear was created on a clean. After allowing the film to dry, it was heat-fixed by being waved over a Bunsen burner’s flame and exposed to a crystal violet reagent. The film was submerged in an iodine solution for one minute before being washed carefully under running water. It was gradually decoloured with an alcohol reagent until no more dye was present. The smear was washed under slowly flowing water after Safranin reagent exposure and examined under a microscope after air drying.

### Antimicrobial susceptibility testing

The National Committee for Clinical Laboratory Standards’ disc diffusion method was used to assess each isolated organism for antimicrobial sensitivity [11]. Ciprofloxacin (CIP), Ampicillin (Amp), Gentamycin (GEN), Cefuroxime (CER), Nitrofurantoin (NIT), Ofloxacin (OFX), Tetracycline (TET), and Cephalexin are among the antibiotics for gram-negative pathogens included in the multi-disc (CEPH).

Antibiotic susceptibility patterns were assessed using the Kirby-Bauer disc diffusion method.

According to the standards of the Clinical Laboratory Standards Institute (2018), the diameter of the zone of inhibition for each antibiotic was measured and classified as resistant, intermediate, or sensitive. The antibiotics used for the susceptibility tests are presented in Table 1.

**Table 1:** List of antibiotics for susceptibility tests

Antibiotics	Disk content (µg)	Resistance (R)	Intermediate (I)	Sensitive (S)
Cefuroxime	30	≤ 14	15–17	≥ 18
Ceftriaxone	30	≤ 19	20–22	≥ 23
Ceftazidime	30	≤ 17	18–20	≥ 21
Cefazolin	30	≤ 19	20–22	≥ 23
Ciprofloxacin	5	≤ 15	16–20	≥ 21
Azithromycin	15	≤ 12	–	≥ 13
Cefoxitin	30	≤ 14	15–17	≥ 18
Trimethoprim	1.25/23.75	≤ 10	11–15	≥ 16
Ampicillin	10	≤ 13	14–16	≥ 17
Chloramphenicol	30	≤ 12	13–17	≥ 18
Tetracycline	30	≤ 11	12–14	≥ 15

### Biochemical testing

This was used to pinpoint the precise organism that was present in the urine specimens. The following biochemical assays were carried out: catalase, coagulase, methyl red, indole, urease, and citrate utilisation.

#### Catalase test

In a test tube, two millimetres of hydrogen peroxide (H_2_O_2_) solution were added. A stock colony of the test organism was selected using a sterile applicator, and it was submerged in the hydrogen peroxide solution. The tube was then examined for bubbles, with active bubbles indicating a positive catalase test and inactive bubbles indicating a negative catalase test (Table 2).

**Table 2:** List of pathogens and their various biochemical test

Biochemical Tests	*Klebsiella* spp	*Klebsiella oxytoca*	*E. coli*	*Acinetobacter baumannii*	*Citrobacter freudii*	*Pseudomonas aeruginosa*
Catalase test	** + **	** + **	** + **	** + **	** + **	** + **
Indole test	** − **	** + **	** + **	** − **	** − **	** − **
Urease test	** + **	** + **	** − **	** − **	** − **	** − **
Methyl red test	** − **	** − **	** + **	** + **	** + **	** − **
Citrate test	** + **	** + **	** − **	** − **	** + **	** + **

#### Citrate utilisation test

The young isolate culture was cultured in slant tubes of Simon citrate agar. Using sterile straight tubes containing buffered glucose, the inoculation was performed by stabbing the medium into the tube. Isolates were carefully introduced into peptone broth. The tubes were incubated at 37 °C for at least 48 h. To 5 mL of the culture, approximately 5 drops of the methyl red reagent were applied. A successful outcome was shown by the immediate appearance of a vivid red colour after the addition of the reagent (Table 2).

#### Indole test

The studied organism was injected into tryptophan- and peptone-containing peptone water. For 48 h, the broth was incubated at 35 °C. After adding 0.5 mL of Kovac’s reagent and shaking the indole, it was analysed. After 10 min, adding Kovac’s reagent (Kovac’s reagent is 4-p-dimethylaminobenzaldehyde), the indole positive develops a red colour layer (Table 2).

#### Methyl red test

This test was used to determine whether the isolates were able to sufficiently create and retain an acid product from the fermentation of glucose. The pink colour was produced when the alkali oxidises the acetyl methyl carbonyl (acetonic) diacetyl (Table 2).

#### Urease test

A tube of urea agar was inoculated with a whole loop of the strain. When the tube was incubated at 37 °C, urease was detected by a change in colour from yellow to red (Table 2).

## Results

### Bacteria isolates

In all, the total number of cases from January to April was 130, the number of positive cases was 42 cases and the rest were negative which were discarded.

### Age distribution of the study population

**Fig. 1:**
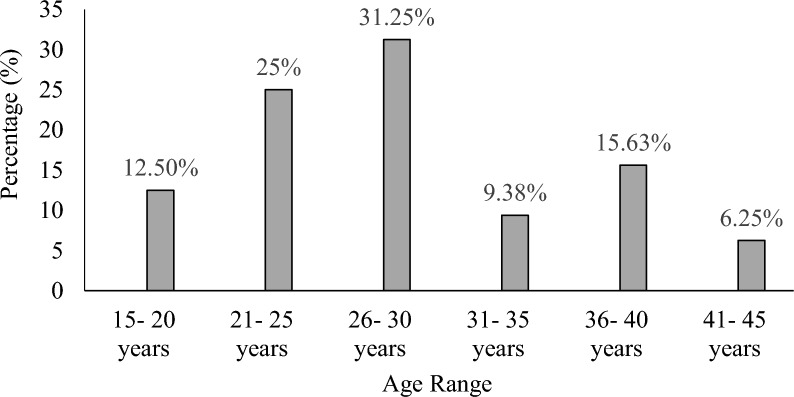
Percentage age distribution of the study population

The study age range was females in their reproductive ages (15–45 years), 26–30 years were 31.25% with the least being 41–45 years. Figure 1 below shows the ages and percentage distribution.

### Bacteria isolate the distribution of positive cases

Six Gram-negatives were isolated including *Klebsiella* spp*, Klebsiella oxytoca, E*. *coli, Acinetobacter baumannii, Pseudomonas aeruginosa, Citrobacter freudii*, and some gram-positives such as *Candida* spp and *Staphylococcus aureus.* The number of positive cases for *Klebsiella* spp was 13, *Klebsiella oxytoca* was 6,* E*. *coli* was 10*, Acinetobacter baumannii* was 3, *Citrobacter freudii* was 1*, Pseudomonas aeruginosa* was 1 and the number of other Gram-positive was 8 (Table 3).

**Table 3:** Bacteria distribution of positive cases without prevalence rate

Bacteria isolate	Positive cases
*Klebsiella* spp	13
*Klebsiella oxytoca*	6
*E*. *coli*	10
*Acinetobacter baumannii*	3
*Citrobacter freudii*	1
*Pseudomonas aeruginosa*	1
Other gram-positives microorganisms	8
Total	42

### Percentage isolates

**Fig. 2:**
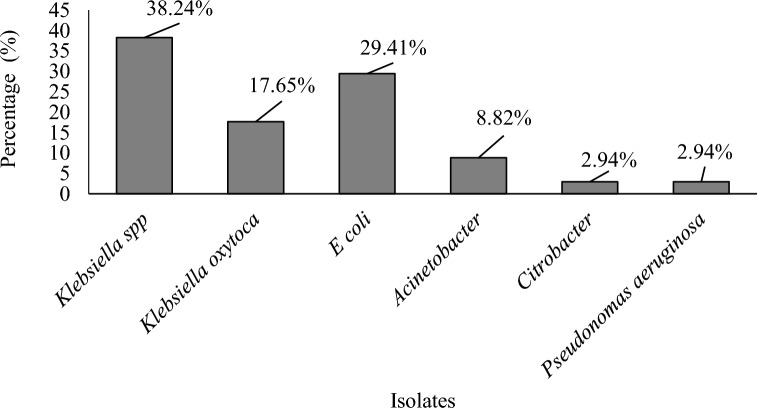
Percentage of isolates

Figure 2 shows the percentages of Gram-negative isolates.

### Prevalence of isolates in urine according to age ranges

*Klebsiella* spp had a prevalence rate of 30.95% followed by *E. coli* (23.81%), *Klebsiella oxytoca* (14.29%), *Acinetobacter baumannii* (7.14%), *Citrobacter freudii* (2.38%), *Pseudomonas aeruginosa* (2.38%) and the other Gram-positive organisms put together had 19.05%. Among the 132 urine samples with both significant and insignificant bacterial growth analysed, six bacterial pathogens were isolated.

### Distribution of isolates according to age ranges

*Klebsiella* spp had a total of 13 positive cases with the highest record of 5 positive cases within the age range 21–25 years with the lowest record from the age range 36–40 and 41–45 years (Table 4). *Klebsiella oxytoca* had a total of 6 positive cases with 2 positive cases being the highest record from the age range of 36–40 years (Table 4). *E. coli* had a total of 10 positive cases with the highest record being 4 positive cases from the age range of 26–30 years*. Acinetobacter baumannii* had a total of 3 positive cases with the highest record being 2 positive cases from the age range of 21–25 years with the least being 1 positive case from the age range of 15–20 years (Table 4)*. Citrobacter freudii* had 1 positive case which was within the age range of 26–30 years. *Pseudomonas aeruginosa* also had only 1 positive case from the age range of 21–25 years. Other Gram-positives such as *Candida* spp and *Staphylococcus aureus* were also recorded during the study.

**Table 4:** Pathogens isolated concerning different age groups

Isolates	15–20	21–25	26–30	31–35	36–40	41–45	Total
*Klebsiella* spp	2	5	4	1	1	–	13
*Klebsiella oxytoca*	1	1	–	1	2	1	6
*E. coli*	2	–	4	1	2	1	10
*Acinetobacter baumannii*	1	2	–	–	–	–	3
*Citrobacter freudii*	–	–	1	–	–	–	1
*Pseudomonas aeruginosa*	–	1	–	–	–	–	1
Other Gram-positives	1	6	1	-	–	–	8

### Antimicrobial susceptibility patterns of isolates

Table 5 shows the antibiotics that were used. Sixteen antibiotics were tested against the isolated organisms.

**Table 5:** Antibiotics tested against the isolated organisms

Antimicrobials	Resistance (R)	Sensitive (S)	Intermediate (I)
Trimethoprim/sulfamethoxazole (SXT)	4	–	–
Nitrofurantion (NIT)	4	4	1
Penicillin (P)	1	–	–
Cefoxitin (FOX)	1	–	–
Cefuroxime (CXM)	18	2	–
Ceftriaxone (CRO)	17	1	–
Levofloxacin (LVX)	12	8	–
Doxycycline (D)	8	–	–
Ceftazidime (CAZ)	17	1	–
Ampicillin (AM)	18	–	–
Cefepime (FEP)	11	–	–
Meropenem (MRP)	10	11	–
Ciprofloxacin (CIP)	19	6	–
Gentamicin (CN)	2	–	–
Erythromycin (E)	1	1	–
Azithromycin (AZM)	1	1	–

### Antimicrobial susceptibility of *Klebsiella* spp

Table 6 shows the antibiotics that were used in testing for the antimicrobial susceptibility of *Klebsiella* spp. *Klebsiella* spp was more resistant to cefuroxime (CXM) (69.23%) and more sensitive to ciprofloxacin (CIP) (38.46%) and meropenem (MRP) (38.46%).

**Table 6:** Antibiotics tested against *Klebsiella* spp

Antibiotics	Sensitive (S)	Resistance (R)
MRP	3 (23.08%)	5 (38.46%)
CIP	3(23.08%)	5 (38.46%)
CN	–	–
AM	–	3 (23.08%)
CAZ	1 (7.69%)	6 (46.15%)
LVX	1(7.69%)	4 (30.77%)
CXM	1 (7.69%)	9 (69.23%)
FEP	–	4 (30.77%)
NIT	2 (15.38%)	1 (7.69%)
SXT	–	2 (15.38%)

### Antimicrobial susceptibility of *Klebsiella oxytoca*

Table 7 shows the antibiotics that were used in testing for the antimicrobial susceptibility of *Klebsiella oxytoca. Klebsiella oxytoca* was showed much resistance to ceftriaxone (100%) and sensitive to meropenem (100%).

**Table 7:** Antibiotics tested against *Klebsiella oxytoca*

Antibiotics	Sensitive (S)	Resistance (R)	Intermediate (I)
MRP	3 (50%)	–	–
LVX	2 (33.33%)	3 (50%)	–
CRO	–	5 (83.33%)	–
AM	–	6(100%)	–
D	–	3(50%)	–
CXM	–	4(66.67%)	–
CIP	1 (16.67%)	4 (66.67%)	–
CAZ	–	2 (33.33%)	–
SXT	–	1 (16.67%)	–
NIT	1 (16.67%)	2 (33.33%)	1(16.67%)
FEP	–	4 (66.67%)	–

#### Antimicrobial susceptibility of *E. coli*

Table 8 shows the antibiotics that were used in testing for the antimicrobial susceptibility of *E. coli. E. coli* showed much resistance in ceftazidime (40%) and was sensitive to meropenem (40%).

**Table 8:** Antibiotics tested against *E. coli*

Antibiotics	Sensitive (S)	Resistance (R)
MRP	4 (40%)	4 (40%)
CN	–	2 (20%)
LVX	2 (20%)	4 (40%)
AM	–	4 (40%)
CRO	1 (10%)	3 (30%)
FEP	–	2 (20%)
CAZ	–	6 (60%)
D	–	3 (30%)
AZM	–	3 (30%)
CXM	–	3 (30%)

#### Antimicrobial susceptibility *of Acinetobacter baumannii*

Table 9 shows the antibiotics that were used for testing the antimicrobial susceptibility of *Acinetobacter baumannii* which was resistant to ciprofloxacin (66.67%), ceftazidime (33.33%), cefuroxime (66.67%), ceftriaxone (33.33%), ampicillin (66.67%), meropenem (33.33%), trimethoprim/sulfamethoxazole (33.33%), nitrofurantin (33.33%), and cefepime (33.33%).

**Table 9:** Antibiotics tested against *Acinetobacter baumannii*

Antibiotics	Sensitive (S)	Resistance (R)
CIP	–	2 (66.67%)
CAZ	–	1 (33.33%)
CXM	–	2 (66.67%)
CRO	–	1 (33.33%)
AM	–	2 (66.67%)
MRP	–	1 (33.33%)
SXT	–	1 (33.33%)
NIT	–	1 (33.33%)
FEP	–	1 (33.33%)

#### Antimicrobial susceptibility of *Citrobacter freudii*

Table 10 shows the antibiotics that were used for testing the antimicrobial susceptibility of *Citrobacter freudii* which was resistant (100%) to ceftazidime, cefepime, ciprofloxacin and levofloxacin.

**Table 10:** Antibiotics tested against *Citrobacter freudii*

Antibiotics	Sensitive (S)	Resistance (R)
CAZ	–	1 (100%)
FEP	–	1 (100%)
CIP	–	1 (100%)
LVX	–	1 (100%)

#### Antimicrobial susceptibility of *Pseudomonas aeruginosa*

Table 11 shows the antibiotics that were used for testing the antimicrobial susceptibility of *Pseudomonas aeruginosa* which was sensitive to meropenem, but resistant to ceftriaxone, ampicillin, doxycycline and levofloxacin.

**Table 11:** Antibiotics tested against *Pseudomonas aeruginosa*

Antibiotics	Sensitive (S)	Resistance (R)
MRP	1 (100%)	–
LVX	1 (100%)	–
CRO	–	1 (100%)
AM	–	1 (100%)
D	–	1 (100%)

## Discussion

The age group 21–30 years (56%) showed the highest number of respondents. This large number of participants in this age category could be due to individuals being sexually active and being in the peak reproductive age range. A high risk of exposure may have resulted from having several sexual partners and poor personal cleanliness. This result was consistent with a study by Singh et al. [38] that found 54.90% of participants were between the ages of 18 and 35. The age range of 41 to 45 years reported the fewest respondents (6.25%). The low turnout of individuals in the age range of 41–45 years may have been caused by improved staff hygiene, which is thought to get better with age and decreased sexual activity, which led to less exposure [32]. Since, older women are more likely to have asymptomatic bacteriuria, which frequently goes away on its own and is not related to morbidity or mortality, so most of them may choose not to seek medical attention [28]. Because of the high population density, neighbouring slum regions, and high rates of urbanisation, it is possible that the prevalence of bacteria among females of reproductive age is related to the participants having several partners for casual sex. The elimination of 56 vaginal natural flora caused by improper use of antibiotics may have decreased vaginal immunity and increased the prevalence of bacteriuria in this study population [16]. Urinary tract infection affects people of both sexes and all age groups [16]. 50% of women experience at least one episode of UTI throughout their lifetime due to anatomical posture, physiological changes, vaginal activity, the use of contraceptives like spermicide and the diaphragm, and a lack of prostatic fluid, which functions as an antibacterial agent [42].

*Klebsiella* spp (38.24%) had the highest percentage followed by *E. coli* (29.41%), *Klebsiella oxytoca* (17.65%), *Acinetobacter* (8.84%) with *Citrobacter freudii* (2.94%) and *Pseudomonas aeruginosa* (2.94%) having the least. Urinary tract infection is a unembellished public health issue with *Klebsiella pneumoniae* causing about 25% of all urinary tract infections that affects people around the world [22]. The associated higher rate of urinary tract infection with uropathogenic *Klebsiella* species has been related with the hypervirulent emergence and antibiotic-resistant strains caused by the antibiotics overuse and misuse in addition to other behavioural practices and sociodemographic of vulnerable individuals [22].

This study *Escherichia coli* is the second highest recorded. It is the most common cause of urinary tract infection causing about 75% of all UTI bacterial cases [41]. Uropathogenic *Klebsiella* species and *E. coli* are the most common cause of urinary tract infections in the healthcare settings and community (Gajdács et al. 2019). Other causative agents that have been reported include *Staphylococcus aureus, Klebsiella pneumoniae*, *Pseudomonas aeruginosa, Proteus mirabilis*, and *Enterococcus faecalis* [41]. These species in the urinary system are successful pathogens, as they possess the pertinent virulence factors mandatory to effectively survive on and stick to the urinary epithelium, cause damage to tissue, and evade immune responses of a host (Gajdács et al., 2019). Similar studies isolated *E. coli* (40.50%), *S. aureus* (14.30%), *S. saprophytics* (9.50%), *K. pneumoniae* (7.10%), *E. faecalis* (7.10%), *S. pyogenes* (7.10%), *K. oxytoca* (4.80%), *Citrobacter* spp (4.80%), *Proteus mirabilis* (2.40%), and *M. morganii* (2.40%) (Yetera et al. 2024). Also, the isolated uropathogen was reported as *Cedecea* (0.60%), *Pseudomonas* (1.70%), *Proteus* (2.80%), *Enterobacte*r (3.40%), *Klebsiella* (21.20%) and *E. coli* (70.40%) (Al-Zahrani et al. 2019).

A higher frequency of bacteria in this study was seen in the age groups of 15–30 years in the 132 urine samples that were examined. The frequency of bacteria may have been higher in the 15 to 30 age group compared to other age groups because women in this age range are at the height of their reproductive potential and are therefore more sexually active, with the majority of them possibly having several partners. An increased risk of exposure could also be caused by poor personal hygiene. It has been determined that certain behavioural characteristics and anatomical or physical anomalies in young women make them more likely to develop UTIs [23]. According to the study, among females, the frequency of bacteriuria considerably decreased as age rose. Between the ages of 41 and 45, there were fewer cases of bacteriuria reported. They are thought to have better personal cleanliness and rarely abuse antibiotics [10]. This result is consistent with a prior study by [38], which found an 8.90% prevalence rate for the age range of 38–48 years.

*Klebsiella* species accounting for 3–20% of cases of urinary tract infections are a common cause [48], Gajdács et al. 2019). In a study showed *Klebsiella* species are the most common bacteria causing urinary tract infections in Dhaka city [48]. Also, *Klebsiella* was found as the second most prevalent uropathogen representing 42.29–56.75% after *Escherichia coli* (Gajdács et al. 2019). The prevalence of *Klebsiella* spp among pregnant women in the current study was 30.95% followed by *E. coli* (23.81%), *Klebsiella oxytoca* (14.29%), *Acinetobacter baumannii* (7.14%), *Citrobacter freudii* (2.38%), *Pseudomonas aeruginosa* (2.38%) and the other gram-positive organisms put together had 19.05%. The UTI prevalence among females in their reproductive age group (15–45 years) in the current study (31.82%) is consistent with studies conducted in Cameroon (31%) (Ngong et al. 2021) and Al Samawa City of Iraq (86%) (Nahab et al. 2022). Nevertheless, this study finding is lower than the UTI prevalence reported from Lebanon (18.0%) (Al Kady et al. 2024), Hawassa, Ethiopia (13.6%) (Yetera et al. 2024), Iran (13.1%) (Rejali and Ahmadi 2019), Kenya (15.7%) [30], Johannesburg (16.8%) [31], Dire Dawa (14%) (Derese et al. 2016), and Addis Ababa (14.9%) [49]. Differences in prevalence could be attributable to factors such as community social habits, sample size, standards of personal hygiene, and educational backgrounds of the participants.

Among the 132 urine samples with both significant and insignificant bacterial growth analysed, six bacterial pathogens were isolated. In this study, the most dominant bacterial pathogen agent isolated from among females in their reproductive age group (15–45 years) was *Klebsiella* spp (30.95%). The second most isolated pathogen was *E. coli* (23.81%). *E. coli* was most common isolated bacteria from some previous studies such as Gonder (49.20%) [9], Hawassa (47.80%) [40], Kenya (44.50%) [30], Somaliland (43.50%) [4], Bangladesh (38.00%) [21] and Dessie (33.30%) [7]. This study’s results are lower compared to some studies reported in Somaliland (43.50%) [4], Kenya (44.50%) [30], Hawassa (47.80%) [40] and Gonder (49.20%) [9]. *Escherichia coli* is the most frequently isolated organism (Wing et al. 2013).

The other bacterial isolates in this study included; *Klebsiella oxytoca*, *Acinetobacter baumannii*, *Citrobacter freudii* and *Pseudomonas aeruginosa*. Similar studies also reported other causative agents of UTI such as *Enterococcus*, *Klebsiella pneumoniae, Staphylococcus, Streptococcus* and *Proteus* species (Wing et al. 2013), *Citrobacter* species, *Enterococcus* species, *Enterobacter clocae, Klebsiella kneumoniae*, *Proteus mirbalis,* and *Pseudomonas aeruginosa* [25].

In this study, the patterns of antimicrobial susceptibility of the recovered isolates were evaluated, and the main isolate *Klebsiella* spp was more resistant to cefuroxime (CXM) (69.23%), *Klebsiella oxytoca* resistant to ceftriaxone (100%)*, E. coli* resistant in ceftazidime (40%), *Acinetobacter baumannii* resistant to ciprofloxacin (66.67%), ceftazidime (33.33%), cefuroxime (66.67%), ceftriaxone (33.33%), ampicillin (66.67%), meropenem (33.33%), trimethoprim/sulfamethoxazole (33.33%), nitrofurantin (33.33%), and cefepime (33.33%) and *Citrobacter freudii* resistant (100%) to ceftazidime, cefepime, ciprofloxacin and levofloxacin. Their effectiveness varied based on the bacterial strains and the antibiotics used. This variation is usually owing to the difference in isolated bacteria and drug-resistant bacterial strains [1]. The high resistance to these antibiotics can be caused by self-medication and antibiotic abuse in hospitals and communities [19]. This result proves the lack of effective or proper antimicrobial practices and infection control strategies in the Northern Region of Ghana. There is evidence of abuse of antibiotics in Ethiopia (Alemayehu Reta and Abeba Mengist 2019). which is most likely the case in Ghana. This, along with a poor surveillance system and the rapid bacteria spread with resistance contributes to the antimicrobial resistance problem [1], Alemayehu Reta and Abeba Mengist 2019).

Ciprofloxacin (CIP), Ampicillin (AM) and Cefuroxime (CXM) showed much resistance whilst the isolates were much more sensitive to Meropenem (MRP) and Levofloxacin (LVX). The Ministry of Health conducted an in vitro susceptibility test on all isolated bacterial pathogens using sixteen antibiotics that are frequently administered as first- and second-line treatments for urinary tract infections. Any antibiotic used to treat an infection must be chosen specifically for each patient based on their allergy history, local medical practices, the prevalence of antibiotic resistance, their accessibility, cost, and level of adherence to the prescribed course of action [34]. The most vulnerable bacterial pathogens to MRP and LVX were isolated strains*. Acinetobacter baumannii* was the only isolated bacterial pathogen resistant to MRP. *Acinetobacter baumannii* is resistant to most commonly used antibiotics.

The second most effective drug was LVX. The isolated bacterial pathogens which were sensitive to LVX include; *Klebsiella spp*, *E. coli, Klebsiella oxytoca* and *Pseudomonas aeruginosa*. *Citrobacter freudii* isolates were resistant to LVX in this study. Gram-negative bacteria are those that lose their crystal violet colour when subjected to the gram-staining procedure. Gram-negative bacteria include, among others, *Escherichia coli*, *Klebsiella* species, *Pseudomonas* species and *Proteus* species. Gram-negative bacteria are non-spore-producing bacilli that proliferate quickly in both aerobic and anaerobic environments on common laboratory media. Some bacteria are naturally resistant to even recently developed anti-bacterial drugs, the majority of pathogens have been found to develop acquired antimicrobial resistance [44]. Antimicrobials have been used to protect the human population from the threat of infectious diseases and are one of the most effective types of chemotherapy (Das and Patra 2017). To reduce drug resistance, antibiotic treatment should start as soon as culture findings are available, and antimicrobial sensitivity testing should be utilised to guide therapy [8].

Antibiotic use has increased over time, resulting in greater drug resistance in microorganisms (Fair and Tor 2014; Laxminarayan and Chaudhury 2016). Antibiotic use has been high, particularly for UTI (Ballesteros-Monrreal et al. 2020). Most UTIs are treated with empirical antibiotics, without antimicrobial susceptibility testing or urine culture (Patel et al. 2019). Such a condition is likely to promote the evolution of resistant species. A study found that more than half of isolates were resistant to standard first-line antibiotics such as ciprofloxacin, ampicillin, and ticarcillin (Mohapatra et al. 2022), while an Iranian investigation found a similar resistance pattern (Yazdanpour et al. 2020). Promoting antimicrobial use during urinary tract infections outpatient treatment is particularly difficult in low-resource settings due to limitations in existing guidelines, diagnostic uncertainties, and rising antimicrobial resistance [18]. Antibiotic overuse for URIs in pregnant women has been reported at rates of more than 96% [18, 37], Zhao et al. 2021), owing to the use of rapid screening methods based on clinical suspicion or instead of microbiological confirmation.

This study has provided the first insight and relevant information on the prevalence and antimicrobial susceptibility of bacteria in the urine of females in their reproductive ages at Tamale Teaching Hospital. The diagnosis of UTIs is crucial for treatment as they spread from one region to another. Therefore, for effective treatment, professionals require some background knowledge, such as the most common bacteria that cause UTIs and their susceptibility to antibiotics. Although the study included individuals in peri-urban areas, it did not conduct a spatial analysis to probably identify important hotspots such as other hospitals, polyclinics, and clinics. Also, the study was conducted within four months; the situation could be different in other months. Future research should consider other hospitals, polyclinics, and clinics in the field.

## Conclusion

The study has revealed that the most common isolate is *Klebsiella* spp among the female in their reproductive ages. This gram-negative organism was shown to be sensitive to the following drugs: Levofloxacin (LVX) and Meropenem (MRP). Gram-negative was high in the age group 21–25 years, followed by the age group 26–30 years and then 15–20 years. To lower the incidence of bacteriuria, public education and awareness campaigns on hygiene are strongly advised.

## Supplementary Information

Additional file 1.

## Data Availability

No datasets were generated or analysed during the current study.
