# Supplementary material for: Prevalence and antimicrobial susceptibility of gram-negative bacteria in the urine of females in their reproductive ages in the Tamale Teaching Hospital
Source: J Health Popul Nutr. 2025 Aug 4;44:276. doi: 10.1186/s41043-025-00853-y (PMC12320360; doi:10.1186/s41043-025-00853-y)
Supplement: Supplementary file 1 — Additional file 1. [file 41043_2025_853_MOESM1_ESM.pdf]

UNIVERSITY FOR DEVELOPMENT STUDIES
Tel: 03720-93382/26634/22078
Email: registrar@uds.edu.gh
Website: www.uds.edu.gh
P. O. Box TL 1350
Tamale, Ghana

Our Ref: UDS/RB/032/21

Your Ref: ....

OFFICE OF THE REGISTRAR

Date: 21ST NOVEMBER, 2021

ABUDU BALLU DUWIEJUAH, RASHIDA IBRAHIM
UNIVERSITY FOR DEVELOPMENT STUDIES, TAMALE, GHANA
AND KEN OSEI, TAMALE TEACHING HOSPITAL,
TAMALE, GHANA

ETHICAL APPROVAL NOTIFICATION

With reference to your request for ethical clearance on the research proposal titled "**Profile and Antimicrobial Susceptibility of Bacteria in the Urine of Females in their Reproductive Ages (15 - 45 Years) in Tamale Teaching Hospital**", I write to inform you that the University for Development Studies Institutional Review Board (UDSIRB) found your proposal including the consent forms to be satisfactory and have duly approved same. The mandatory period for the approval is six (6) months, starting from 21st November, 2021 to 21st April, 2022.

Subject to this approval, you are please required to observe the following conditions:

1. That the anonymity of the respondents shall be guaranteed as mentioned in the consent forms.
2. That you will acknowledge the source of the data collected in any publication related to this research.
3. That you will submit a field report and a copy of the research report to the UDSIRB.
4. That you may apply to the UDSIRB for any amendments relating to recruiting methods, informed consent procedures, study design and research personnel.
5. That you will strictly abide by the code of conduct of this University.

Please do not hesitate to refer any issue (s) that you may deem necessary for the attention of the Board.

Thank you.

Prof. Nafiu Amidu
Chairman, UDSIRB
Cc: file

CamScanner
